# Supplementary material for: Induced Mutation in GmCOP1b Enhances the Performance of Soybean under Dense Planting Conditions
Source: Int J Mol Sci. 2022 May 12;23(10):5394. doi: 10.3390/ijms23105394 (PMC9141786; doi:10.3390/ijms23105394)
Supplement: Supplementary file 1 [file ijms-23-05394-s001.zip › ijms-1699593-supplementary.pdf]

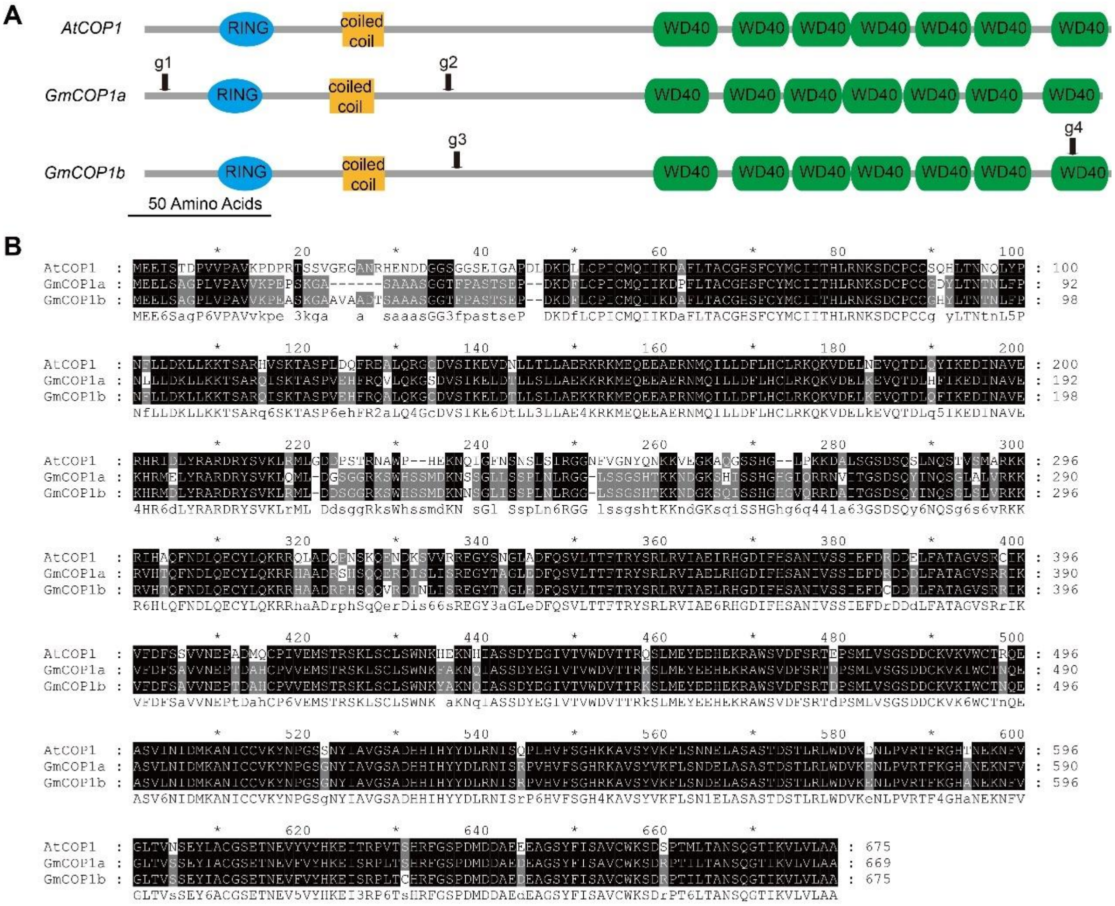

Figure S1. Structure and sequence alignment of COP1 proteins in Arabidopsis and soybean

(**A**) Schematic diagram of AtCOP1, GmCOP1a, and GmCOP1b proteins. g1 to g4 indicate the editing sites. Scale bar = 50 amino acids. (**B**) Protein sequence alignment of AtCOP1, GmCOP1a, and GmCOP1b.

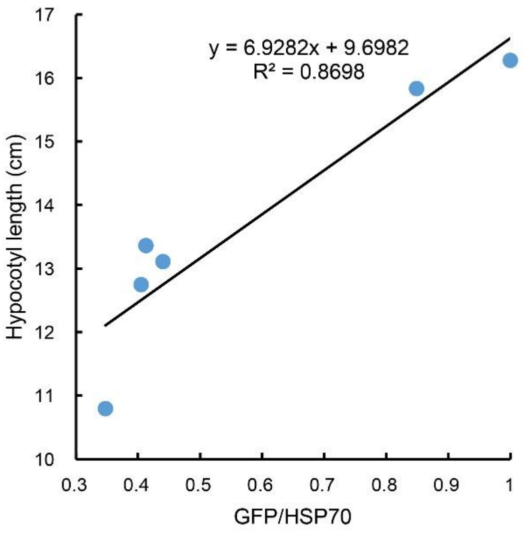

Figure S2. Correlation analysis between the hypocotyl length and protein level of indicated lines in Figure 1B.

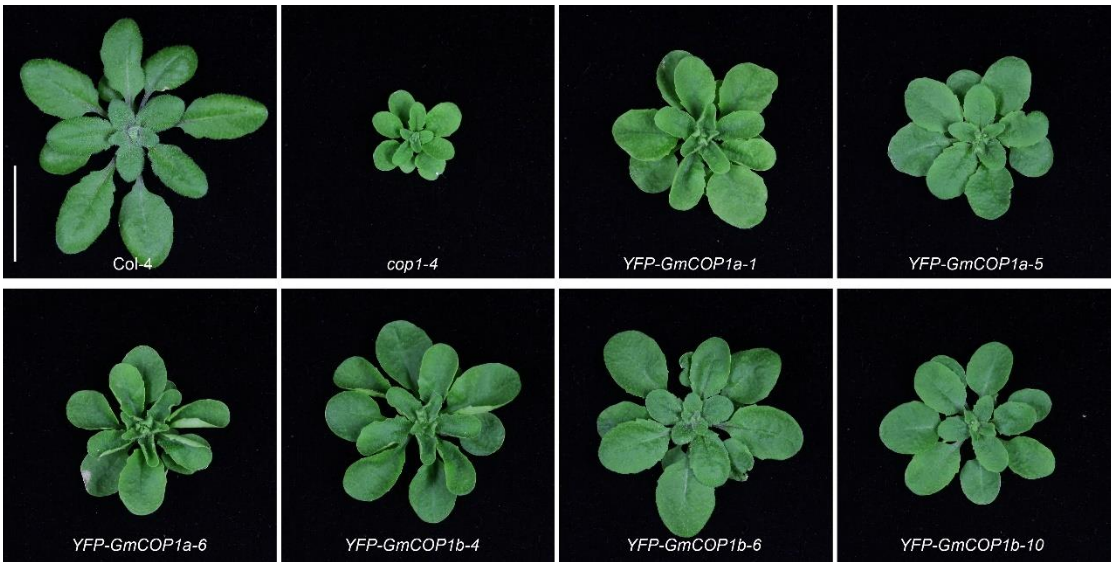

Figure S3. Representative images of indicated lines at vegetative stage

Plants were grown under long-day (16 h light/8 h dark) conditions for 28 days. Scale bar = 2 cm.

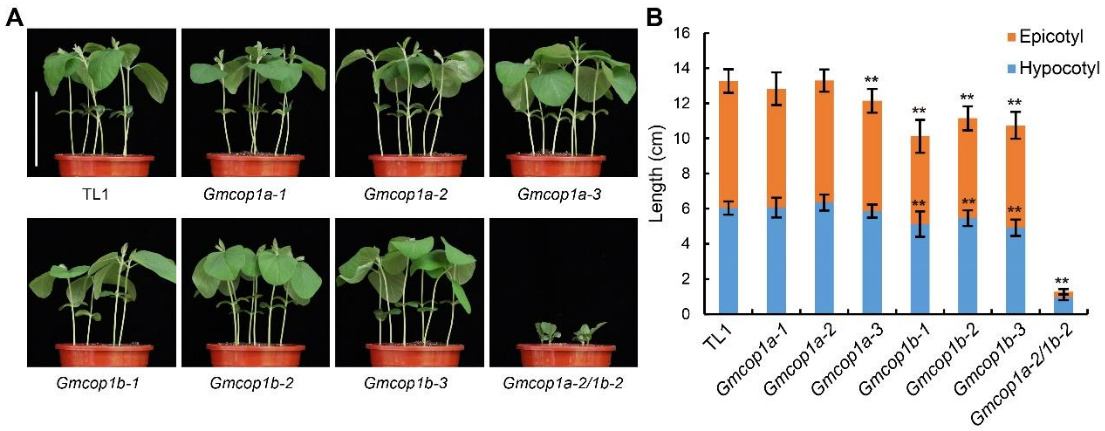

Figure S4. Seedling status of indicated *Gmcop1* mutant lines grown under long-day conditions (**A**) Representative images of different mutants grown under long-day (16 h light/8 h dark) conditions for 10 days. Scale bar = 10 cm. (**B**) Statistical analysis of hypocotyl and epicotyl lengths in (**A**). The values represent the means ± SD (n ≥ 10), asterisks indicate significant differences compared to TL1 by Student's *t*-test (** $P < 0.01$).

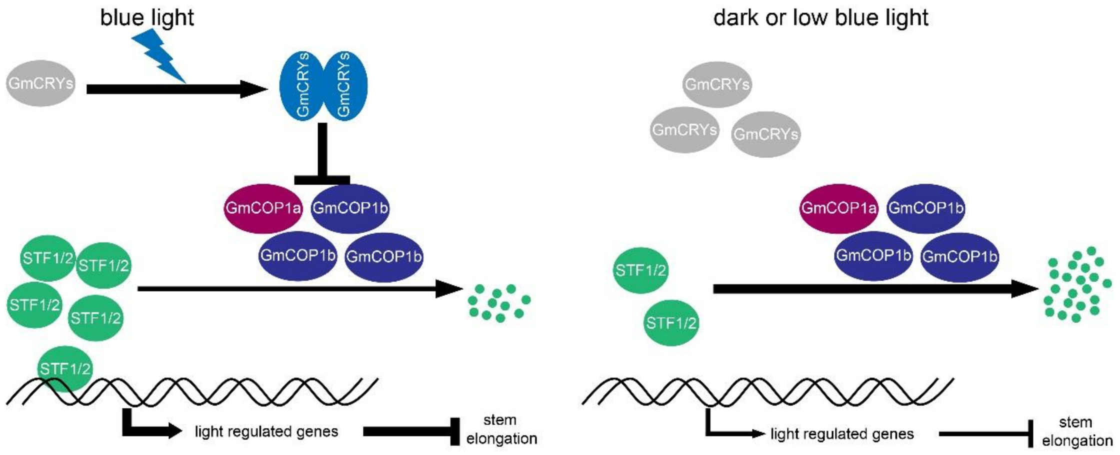

Figure S5. A model for the regulation of stem elongation by GmCOP1s in soybean under dark or LBL conditions

Under light conditions, light-activated GmCRYs inhibit the activity of GmCOP1s, promote the accumulation of STF1/2 proteins which regulate the transcriptions of downstream light-regulated genes to inhibit soybean stem elongation. In the dark or LBL conditions, GmCRYs are deactivated and release the activity of GmCOP1s to degrade the STF1/2 proteins. Therefore, GmCOP1s negatively regulate the expression of light-regulated genes and promote stem elongation.

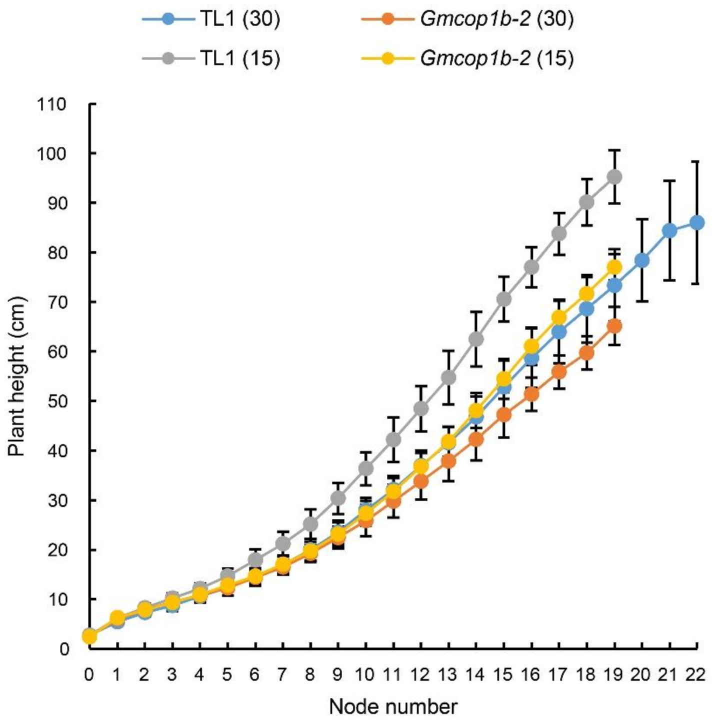

Figure S6. Statistical analysis of each internode length of indicated lines under different planting density in field

The values represent the means ± SD ($n \geq 8$). Numbers in the brackets indicate the plant spacing, the unit is cm.

Table S1. Primer sequences used in this study

**Overexpression plasmids**	primer sequence (5'-3')
YFP-GmCOP1a-F	GTGGGGACAAGTTTGTACAAAAAAGCAGGCTTCATGGAAGAGCTCTCAGCGGG
YFP-GmCOP1a-R	GTGGGGACCACTTTGTACAAGAAAGCTGGGTCAGCTGCAAGCACCAGCACTT
YFP-GmCOP1b-F	GTGGGGACAAGTTTGTACAAAAAAGCAGGCTTCATGGAAGAGCTCTCCGCGGG
YFP-GmCOP1b-R	GTGGGGACCACTTTGTACAAGAAAGCTGGGTCAGCTGCAAGCACCAGCACTT
**CRISPR plasmids**	primer sequence (5'-3')
GmU6-F	GCTTAGGCCTTCTAGAAAAATAAATGGTAAAATGTCAA
GmU6-R	AATCCATGTGGTGGCACATTTC
S1-sgRNA-F	AATGTGCCACCACATGGATTGGGTTCAGGTTTGACGACGGGTTTTAGAGCTAGAAATAGCAA
S2-sgRNA-F	AATGTGCCACCACATGGATTGTGCAGATGCTTGACGGTTCGTTTTAGAGCTAGAAATAGCAA
S3-sgRNA-F	AATGTGCCACCACATGGATTGTACGGATGCTTGACGACTCGTTTTAGAGCTAGAAATAGCAA
S4-sgRNA-F	AATGTGCCACCACATGGATTGCTTCATTAGTGCTGTATGCGTTTTAGAGCTAGAAATAGCAA
scaffold-R	TCGGCAACGCGTTCTAGAAAAAAAAGCACCGACTCGGT
**Genotyping**	primer sequence (5'-3')
S1-F	CCCCAATTCCGAAGCAAA
S1-R	GCAACAAGTTAGGGAAGAGGT
S2-F	GTACAAACTGATCTCCACTTTAT
S2-R	GCACCCTCTTCTTTCTAACT

S3-F	TGGTGTTTCGTGCTTTC
S3-R	GCAATATGGCATCTGCT
S4-F	TGTGGCAGTGAAACAAAT
S4-R	GTTCAGTCCCTTAAATAGGT
BAR-F	TCCGCAGCCATTAACGACTT
BAR-R	ACAGATAAAGCCACGCACATT
CAS9-F	CAGCTCGTCCAAACCTAC
CAS9-R	CTGTGCCATCCATCTTCT
GmU6-sgRNA-F	GCGGTGTCATCTATGTTACTA
GmU6-sgRNA-R	TTCAAGTTGATAACGGACTA
**qPCR**	primer sequence (5’-3’)
GmCOP1a-qF	GCGATTGCCCTTGCTGCGGCG
GmCOP1a-qR	ATACCTGCCGAAAATGTTCG
GmCOP1b-qF	GCGATTGCCCTTGCTGCGGCC
GmCOP1b-qR	ATGCCTGCCGGAAATGTTCC
GmACT11-qF	ATCTTGACTGAGCGTGGTTATTCC
GmACT11-qR	GCTGGTCCTGGCTGTCTCC
